# Lithostratigraphic and magnetostratigraphic data from late Cenozoic glacial and proglacial sequences underlying the Altiplano at La Paz, Bolivia

**DOI:** 10.1016/j.dib.2018.05.038

**Published:** 2018-05-19

**Authors:** Nicholas J. Roberts, René W. Barendregt, John J. Clague

**Affiliations:** a Department of Earth Sciences, Simon Fraser University, Burnaby, Canada V5A 1S6; b Department of Geography, University of Lethbridge, Lethbridge, Canada T1K 3M4

**Keywords:** Plio-Pleistocene transition, Mid-Piacenzian warm period, Glacial stratigraphy, South America, Central Andes, Altiplano, Magnetostratigraphy, Detrital remanent magnetization, Magnetic susceptibility

## Abstract

We provide lithostratigraphic and magnetostratigraphic data derived from a Plio-Pleistocene continental sediment sequence underlying the Altiplano plateau at La Paz, Bolivia. The record comprises six sections along the upper Río La Paz valley, totaling over one kilometre of exposure and forming a ~20-km transect oblique to the adjacent Cordillera Real. Lithostratigraphic characterization includes lithologic and stratigraphic descriptions of units and their contacts. We targeted gravel and diamicton units for paleomagnetic sampling to address gaps in the only previous magnetostratigraphic study from this area. Paleomagnetic data – magnetic susceptibility and primary remanent magnetization revealed by progressive alternating field demagnetization – are derived from 808 individually oriented samples of flat-lying, fine-grained sediments. The datasets enable characterization of paleo-surfaces within the sequence, correlation between stratigraphic sections, and differentiation of asynchronous, but lithologically similar units. Correlation of the composite polarity sequence to the geomagnetic polarity time scale supports a range of late Cenozoic paleoenvironmental topics of regional to global importance: the number and ages of early glaciations in the tropical Andes; interhemispheric comparison of paleoclimate during the Plio-Pleistocene climatic transition; timing of and controls on inter-American faunal exchange; and the variability of Earth's paleomagnetic field.

**Specifications Table**

**Table:** 

Subject area	Geology
More specific subject area	Plio-Pleistocene tropical glaciation, landscape evolution, and paleoclimate
Type of data	Tables, figures
How data was acquired	In-field lithostratigraphic characterization; Survey; Sapphire Instruments SI-2B magnetic susceptibility meter; AGICO JR-6A spinner magnetometer; ASC Scientific D-2000 alternating-field demagnetizer
Data format	Raw and analyzed
Experimental factors	Samples were dried then stored in a magnetic shield prior to and between magnetic measurements
Experimental features	Lithostratigraphic characterization includes texture, structure, lithology, colour, clast size and shape, sorting, weathering features, diamicton fabric, and the nature of contacts. We collected groups of typically six individually oriented cylindrical samples from 124 sample locations and processed them at the University of Lethbridge, Alberta, Canada. Magnetic susceptibility was measured with a Sapphire Instruments SI-2B magnetic susceptibility meter. Remanent magnetization was measured with an AGICO JR-6A spinner magnetometer prior to and after stepwise demagnetization using an ASC Scientific D-2000 alternating-field demagnetizer (4 to 16 steps at 2.5–30 mT spacing). Remanence directions were determined for most samples by principal component analysis and for a small number of samples (<2%) by the intersection of great circles. We calculated remanence directions of samples and mean remanence directions by group, stratigraphic unit, and polarity using AGICO's Remasoft v. 3.0.
Data source location	City of La Paz, Department of La Paz, Bolivia (16°30′ S, 68°9′ W)
Data accessibility	Data are within this article and in related references
Related research article	Roberts et al. (2017, 2018)

**Value of the data**

• Provides detailed lithostratigraphic and magnetostratigraphic records of the earliest known tropical glaciation in the Cenozoic Era.
• Enables comparison with global records of paleoenvironmental change during the Plio-Pleistocene climatic transition.
• Presents a detailed record supporting magnetostratigraphic comparison with late Cenozoic sequences underlying other parts of the Altiplano plateau.
• Provides detailed chronostratigraphic constraints of paleoenvironmental change and spatio-temporal variability of land mammal assemblages related to biotic exchange between the Americas.
• Contributes to the growing paleogeomagnetic record for central South America.

## 1 Data

Geologic sequences underlying the Altiplano plateau in the South American Andes provide extensive, but underexplored records of late Cenozoic continental paleoenvironments. Due to the Altiplano's long history as an internally drained basin [1], [2], its sequence of up to 12 km of Tertiary sediments is relatively complete [3]. The low-energy depositional environments represented by many units [3], [4], [5] makes these sediments suitable recorders of variations of the ancient geomagnetic field on a wide range of time scales [6], as demonstrated by the results of the small number of paleomagnetic investigations in the region [7], [8], [9], [10].

Despite the importance of records from the sub-Altiplano fill sequence, their ages are generally poorly constrained. Current chronologic control is based largely on radiometric dating of volcanic beds within the sequence [11], [12], [13], but many of the ages are unreliable [14]. Magnetostratigraphy at a few localities across the Altiplano constrains ages of non-volcanic units as well as their accumulation rates [7], [8], [9], [10].

**Fig. 1:**
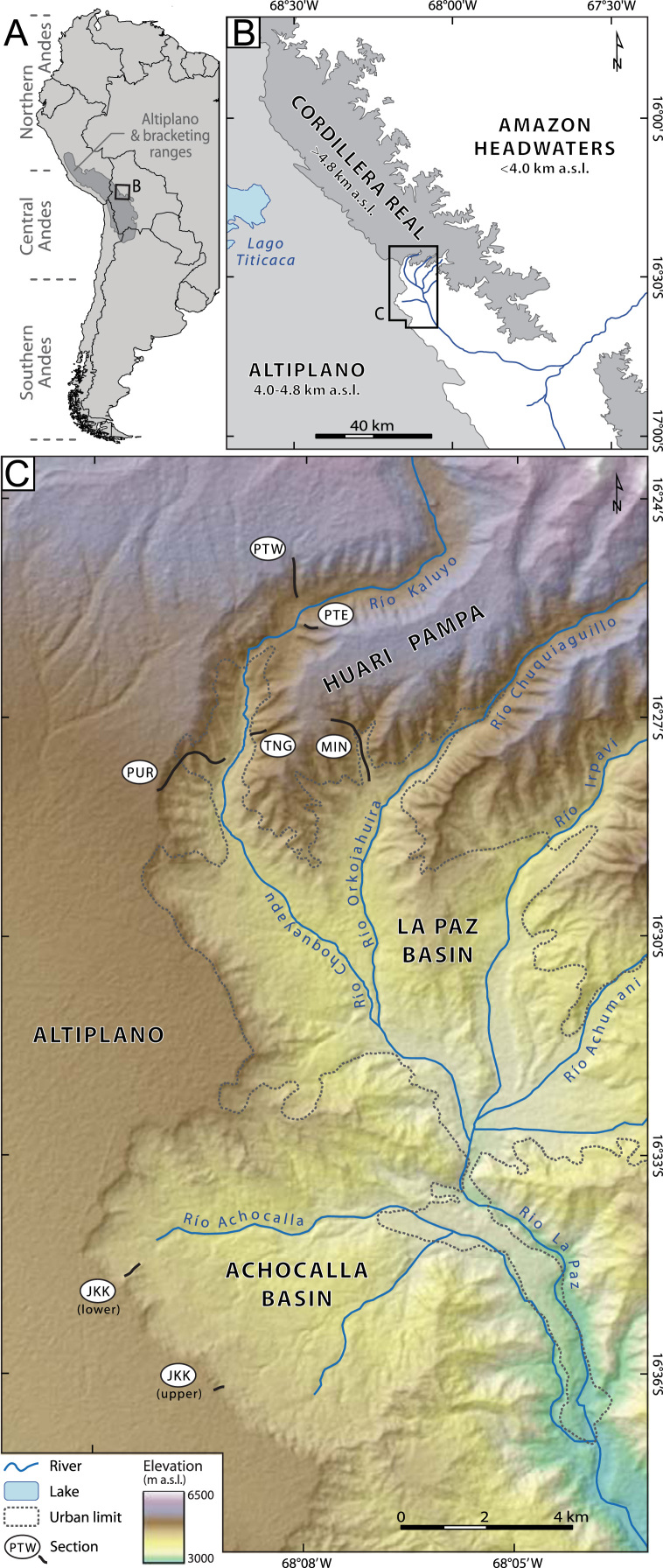
Location map. A. Extent of the Altiplano plateau within South America. B**.** Physiographic setting of the Cordillera Real and adjacent Altiplano margin. C. Locations of lithostratigraphic and magnetostratigraphic sections. Stratigraphic sections mentioned in the text are: PWT, Patapatani West; PTE, Patapatani East; TNG, Tangani; MIN, Minasa; PUR, Purapura; and JKT, Jacha Kkota. Terrain is from the ASTER GDEM 2 produced by METI and NASA.

Here we present chronostratigraphic data from the upper part of the fill sequence at La Paz, Bolivia, where it is extensively exposed. The data come from six sections within and adjacent to the city of La Paz (Fig. 1 and Table 1). Our lithostratigraphic descriptions and magnetostratigraphic data, respectively, build on the Plio-Pleistocene stratigraphic framework developed by previous workers [5], [15], [16], [17], [18], [19], [20], [21] and greatly expand upon the only previous paleomagnetic study in the area [8]. The data provide new insights into several aspects of the Altiplano and adjacent Cordillera Real area during the late Pliocene and Early Pleistocene [24]. Specifically, these data can be used to: demonstrate facies relationships of coeval units fining away from the high cordillera; constrain the number and ages of recurrent Pliocene and Early Pleistocene glaciations of the tropical Andes; revise the ages of several gravel sequences; quantity rates of sediment accumulation, the spatial variability of which help to characterize syndepositional tectonism; constrain the onset of incision of the local Altiplano surface and thus the approximate time of drainage capture of the eastern Altiplano by headwaters of the Amazon River system; and compare the timing of environmental change related to early glaciation of the Central Andes with patterns of Plio-Pleistocene faunal evolution and dispersal in the Americas, including occasional migrations leading up to the Great American Biotic Interchange.

**Table 1:** Summary of stratigraphic sections.

**Section**	**Section height (m)**		**Sample sites**	**Average sample spacing (m)**	**Samples**			**Remanence calculation**	
	Total^a^	Sampled^b^			Total	Useful^c^	Used^c^	PCA^d^	GC^e^
Patapatani West	232	229	47	4.9	328	284	251	251	0
Patapatani East	50	36.5	10	3.7	66	48	41	41	0
Tangani	250	190.5	0	6.4	193	181	160	160	0
Minasa	410	410	14	29.3	84	71	61	61	0
Purapura	250	91	14	6.5	83	70	66	60	6
Jacha Kkota	175	59	9	6.6	54	53	45	41	4
Overall	1367	1016	94	10.8	808	707	624	614	10

a Includes covered slopes and exposures that were not described.

b Includes only described, sampled exposures.

c Useful samples are of sufficient quality to enable identification of polarity, but in some cases yield low-precision remanence directions or remanence directions that are statistical outliers with respect to other samples within the same group. Used samples are those considered in statistical analysis, and do not include samples yielding low-precision remanence directions or remanence directions that are statistical outliers with respect to other samples within the same group.

d Primary remanence directions determined by Principal Component Analysis (PCA) for samples included in overall statistics (Fig. 3A–C and Table 1 of Ref. [14]) and for mean directional data by stratigraphic section (Fig. 3D and Table 1 of Ref. [14]).

e Primary remanence directions determined by intersection of great circles (GC) used for group mean directional data (Fig. 3D and Table 1 of Ref. [14]), but not in overall statistics of entire sample collection (Fig. 3A–C and Table 1 of Ref. [14]).

### 1.1 Section details

#### 1.1.1 Patapatani West

##### 1.1.1.1 Location

The Patapatani West section (16° 25.49′ S, 68° 08.03′ W, 232 m in height; Table 2; Figs. 5 and 6A of Ref. [14]) is located on the west bank of Río Kaluyo, where it curves east above the Limanpata landslide. It is the farthest upstream exposure in the Río Kaluyo/Choqueyapu valley (Fig. 1C) and consists of two exposures, each containing a 10-m-thick tuff at 4260–4270 m a.s.l. The upper exposure (137 m) extends from the base of the Chijini Tuff to the Altiplano surface and is exposed in a gully entering the west side of Quebrada Aquatiña. The lower part of the exposure [22] was uncovered during recent (ca. 2007) roadwork just downvalley of Quebrada Aquatiña. The top of this lower section aligns with Dobrovolny's [5], [18] 7-m type section of the Patapatani Drift on the opposite side of the valley and thus greatly extends exposure of the Patapatani Drift.

Nineteen metres of the lower exposure are covered by spoil dumped downslope during road construction. A 3-m-high exposure on the west side of Quebrada Aquatiña, which does not appear to have slumped [22], provides the only details on stratigraphy and paleomagnetism in this largely covered zone (Fig. 6A of Ref. [14]). The sequence below the base of the exposed section (4165 m a.s.l.) is buried beneath Late Pleistocene and Holocene glacial and colluvial deposits down to Río Kaluyo (4125 m a.s.l.).

##### 1.1.1.2 Stratigraphy

**Table:** 

**Unit**	**Description**^**a**^	**Thickness (m)**
20	Poorly sorted, clast-supported, pebble-cobble gravel	1
	*Strong paleosol*^b^	
19	Massive to weakly stratified, matrix-supported, pebble-cobble-boulder diamicton	17
18	Weakly stratified, clast-supported, pebble-cobble-boulder gravel (10% granitic clasts)	
	(unit 17+unit 18)	27
17	Weakly stratified, clast-supported, pebble-cobble-boulder gravel (10% granitic clasts); sharp wavy basal contact with incorporated clasts from underlying unit	
16	Weakly stratified, clast-supported to clast-supported, pebble-cobble-boulder gravel (90% granitic clasts)	10
	*Weak paleosol*^b^	
15	Massive, matrix-supported, pebble-cobble-boulder diamicton	11
	*Strong paleosol*^b^	
14	Massive, matrix-supported, pebble-cobble-boulder diamicton	4.5
	*Strong paleosol*^b^	
13	Weakly stratified, matrix-supported diamicton grading to gravel	2.5
12	Massive, matrix-supported, pebble-cobble-boulder diamicton	6
	*Weak paleosol*^b^	
11	Massive to weakly stratified, matrix-supported, pebble-cobble-boulder diamicton^c^	48
10	Rhyolitic tuff with rare granite and argillite pebbles; locally faulted	10
9	Massive to very weakly stratified, matrix-supported, pebble-cobble-boulder diamicton (~90% granitic clasts)^c^	5
	*Strong paleosol*^b^	
8	Massive, matrix-supported, pebble-cobble-boulder diamicton (~90% granitic clasts)^c^	4
	*Strong paleosol*^b^	
7	Massive, matrix-supported, pebble-cobble-boulder diamicton (~90% granitic clasts)^c^	8
	*Strong paleosol*^b^	
6	Massive, matrix-supported, pebble-cobble-boulder diamicton with rare sandy silt	
	lenses (~50% granite clasts)^c^	20
	*Weak paleosol*^b^	
5	Massive, matrix-supported, pebble-cobble-boulder diamicton (~50% granitic clasts)^c^	>13
–	Covered	5.5
4	Massive, matrix-supported, pebble-cobble-boulder diamicton (~50% granitic clasts)	>3
–	Covered	13.5
3	Massive, matrix-supported, pebble-cobble-boulder diamicton (~50% granitic clasts)	>15
2	Inclined stratified matrix- supported diamicton with rare silt beds (<10% granitic clasts)^c^	6
1	Weakly stratified, poorly sorted, clast-supported, pebble-cobble gravel with some striated clasts (<10% granitic clasts)	2

^a^All diamicton units contain striated and faceted clasts; most clasts are subrounded to subangular. Most clasts in gravel units are rounded to subrounded.

^b^The paleosols are classified as either strong or weak, depending on the degree of development of soil horizons and pedogenic structure. Strong paleosols are those with B horizons thicker than 50 cm (e.g. Fig. 5C of Ref. [14]) and pedons coated with clay (e.g. Fig. 5I of Ref. [14]). In contrast, weak paleosols are those with B horizons thinner than 30 cm, and little or no clay translocation and pedon formation.

^c^See Fig. 2 for clast fabric.

#### 1.1.2 Patapatani East

##### 1.1.2.1 Location

The Patapatani East section (16° 25.89′ S, 68° 08.06′ W, 50 m in height; Table 3; Fig. 6B of Ref. 14) is located between ~4060 and 4110 m a.s.l., low on the east slope of the Río Kaluyo valley [28], ~1 km south of the Patapatani West section (Fig. 1C). Dobrovolny [5], [18] describes the upper ~25 m of this section and assigns the diamicton below the tuff to the Patapatani Drift. A road cut created in 2003 or 2004 forms the lower 10 m of the Patapatani East section, 15 m below and 40 m southwest of the base of Dobrovolny's [5], [18] natural exposure of the Patapatani Drift. Colluvial deposits cover the steep slope between the natural exposure and the road cut.

##### 1.1.2.2 Stratigraphy

**Table:** 

**Unit**	**Description**^**a**^	**Thickness (m)**
8	Weakly stratified, matrix-supported, pebble-cobble-boulder diamicton	1
7	Horizontally stratified, clast-supported, pebble-cobble gravel with some striated clasts	1
6	Rhyolitic tuff (^40^Ar/^30^Ar step-heating on sanidine biotite recovered from a ~2-kg bulk sample yields an age of 2.74±0.04 Ma; Roberts et al. [14])	5
5	Massive, matrix-supported, pebble-cobble-boulder diamicton (80% granitic clasts)	3
4	Massive, matrix-supported, pebble-cobble-boulder diamicton	1.5
	*Strong paleosol*^b^	
3	Massive, matrix-supported, pebble-cobble-boulder diamicton (60% granitic clasts)^c^	>1.5
–	Covered	15
2	Poorly sorted, very weakly stratified, clast-supported, pebble-cobble-boulder gravel with uncommon contorted silt lenses	>3.5
1	Massive, clast-supported, pebble-cobble diamicton with massive silt lenses near upper contact	3.5

^a^All diamicton units contain striated and faceted clasts; most clasts are subrounded to subangular. Most clasts in gravel units are rounded to subrounded.

^b^The paleosols are classified as either strong or weak, depending on the degree of development of soil horizons and pedogenic structure. Strong paleosols are those with B horizons thicker than 50 cm (e.g. Fig. 5C of Ref. [14]) and pedons coated with clay (e.g. Fig. 5I of Ref. [14]). In contrast, weak paleosols are those with B horizons thinner than 30 cm, and little or no clay translocation and pedon formation.

^c^See Fig. 2 for clast fabric.

#### 1.1.3 Tangani

##### 1.1.3.1 Location

The Tangani section (16° 27.33′ S, 68° 08.78′ W, 250 m in height; Table 4; Fig. 6C of Ref. [14]) is located in Quebrada Capellani, a deeply incised network of gullies on the east bank of Río Choqueyapu, 0.5 km upstream from the hairpin curve on the autopista (Fig. 1C). The uppermost 80 m of the main section are exposed only in vertical inaccessible cliffs; exposures in branches of Quebrada Tangani, ~300 m to the southeast, provide access to units 10–12, which we correlate to the upper part of the Tangani section by elevation and by a thick, laterally persistent silt bed 185 m above the base of the main section.

##### 1.1.3.2 Stratigraphy

**Table:** 

**Unit**	**Description**^**a**^	**Thickness (m)**
13	Gently dipping, stratified, contorted clast-supported gravel (<10% granitic clasts) with a silty sand matrix; unit 13 cuts across underlying units, parallel to valley slope and thus differs in thickness across the exposure	~6
12	Weakly stratified, clast-supported, pebble-cobble-boulder gravel (>80% granitic clasts) with rare laterally extensive silt beds	62
11	Weakly stratified, clast-supported, pebble-cobble-boulder gravel (>80% granitic clasts); location of lower contact is uncertain	~5
10	Weakly stratified, clast-supported, pebble-cobble-boulder gravel (>80% granitic clasts); location of upper contact is uncertain	~35
	*Strong paleosol*^b^	
9	Weakly stratified, clast-supported, pebble-cobble-boulder gravel (>80% granitic clasts)	13
	*Strong paleosol*^b^	
8	Weakly stratified, matrix-supported pebble-cobble diamicton (>60% granitic clasts)	7
	*Strong paleosol*^b^	
7	Weakly stratified, matrix-supported, pebble-cobble diamicton (>60% granitic clasts)	5.5
6	Sub-horizontally stratified, pebble-cobble-boulder gravel (>80% granitic) with numerous silt lenses; gravel ranges from matrix- to clast-supported	14
	*Strong paleosol*^b^	
5	Weakly stratified, matrix-supported, pebble-cobble diamicton (~50% granitic clasts); grades upward into weakly stratified gravel then silt	7
	*Weak paleosol*^b^	
4	Massive, matrix-supported, pebble-cobble-boulder diamicton; largest clasts are granitic (>60% granitic clasts)	79
3	Weakly stratified, clast-supported, pebble-cobble-boulder gravel (>80% granitic clasts)	3.5
2	Massive, matrix-supported, pebble-cobble-boulder diamicton (>50% granitic clasts)	3.5
1	Horizontally stratified, clast-supported, pebble-cobble gravel (>80% granitic clasts)	8

^a^All diamicton units contain striated and faceted clasts; most clasts are subrounded to subangular. Most clasts in gravel units are rounded to subrounded.

^b^The paleosols are classified as either strong or weak, depending on the degree of development of soil horizons and pedogenic structure. Strong paleosols are those with B horizons thicker than 50 cm (e.g. Fig. 5C of Ref. [14]) and pedons coated with clay (e.g. Fig. 5I of Ref. [14]). In contrast, weak paleosols are those with B horizons thinner than 30 cm, and little or no clay translocation and pedon formation.

#### 1.1.4 Minasa

##### 1.1.4.1 Location

The Minasa section (16° 27.85′ S, 68° 07.11′ W, 410 m in height; Table 5; Fig. 6D of Ref. [14]) is located along Río Minasa, a west-bank tributary of Río Orkojahuira in barrio Villa El Carmen (Fig. 1C). It extends from Puente Colonial (~3910 m a.s.l.) to Huari Pampa (4320 m a.s.l.), an undeveloped section of the Altiplano surface between ríos Kaluyo/Choqueyapu and Chuquiaguillo/Orkojahuira. Low natural exposures and road cuts along the south bank of Río Minasa form the lower half of the section. High natural exposures along the steep westernmost gully provide continuous exposure of the upper half of the section. Río Minasa follows the trace of a high-angle fault, but there is no obvious displacement of the Huari Pampa surface along this structure.

##### 1.1.4.2 Stratigraphy

**Table:** 

**Unit**	**Description**^**a**^	**Thickness (m)**
	*Strong paleosol*^b^	
15	Weakly stratified, matrix-supported diamicton with thin basal zone of poorly sorted pebble-cobble gravel (10% granitic clasts)	4
	*Strong paleosol*^b^	
14	Weakly stratified, matrix-supported diamicton with thin basal zone of poorly sorted pebble-cobble gravel (10% granitic clasts)	3.5
13	Weakly stratified, matrix-supported, pebble-cobble diamicton with thin basal zone of poorly sorted pebble-cobble gravel (10% granitic clasts)	11
12	Weakly stratified, matrix-supported, pebble-cobble-boulder diamicton with thin basal zone of poorly sorted pebble-cobble gravel (10% granitic clasts)	16.5
	*Strong paleosol*^b^	
11	Weakly stratified, clast-supported, pebble-cobble gravel	7
10	Weakly stratified, matrix-supported diamicton with thin basal zone of poorly sorted pebble-cobble gravel (10% granitic clasts)	4
9	Weakly stratified, matrix-supported, pebble-cobble diamicton with thin basal zone of poorly sorted pebble-cobble gravel (10% granitic clasts)	3
8	Massive matrix-supported diamicton; includes a pebble-cobble gravel bed	8.5
7	Horizontally stratified, clast-supported, pebble-cobble gravel	6.5
6	Weakly stratified, matrix-supported, pebble-cobble-boulder diamicton	15
5	Horizontally stratified, poorly sorted, clast-supported, pebble-cobble-boulder gravel (<20% granitic clasts)	8
4	Matrix-supported, weakly stratified, pebble-cobble diamicton (<20% granitic clasts)	6
3	Weakly stratified, poorly sorted, clast-supported, pebble-cobble-boulder gravel (granitic	
	clast content decreases from >50% in the lower part of unit to <20% in the upper part of the unit)	173
	*Strong paleosol*^b^	
2	Weakly stratified, poorly sorted, clast-supported, multi-lithic gravel, coarsening upward from pebble-cobble to cobble-boulder (granite content increases in the upper part of the unit from <20% to >50%); 10-m-thick zone near the base of the unit is covered	134
1	Rhyolitic tuff	10

^a^All diamicton units contain striated and faceted clasts; most clasts are subrounded to subangular. Most clasts in gravel units are rounded to subrounded.

^b^The paleosols are classified as either strong or weak, depending on the degree of development of soil horizons and pedogenic structure. Strong paleosols are those with B horizons thicker than 50 cm (e.g. Fig. 5C of Ref. [14]) and pedons coated with clay (e.g. Fig. 5I of Ref. [14]). In contrast, weak paleosols are those with B horizons thinner than 30 cm, and little or no clay translocation and pedon formation.

#### 1.1.5 Purapura

##### 1.1.5.1 Location

The Purapura section (16° 27.74′ S, 68° 09.23′ W, 250 m in height; Table 6; Fig. 6E of Ref. [14]) mostly follows the old railway ascending to the Altiplano along the west slope of the Río Choqueyapu valley (Fig. 1C). The lower part of the section crosses this slope obliquely between quebradas Jacha and Pantisirca along the old railway route where it passes under the aqueduct. The upper part of the section follows the railway route on its final (60-m elevation gain) approach to the plateau. This section roughly coincides with the ‘Pura Pura’ (Aqueducto) section of Bles et al. [19] and Ballivián et al. [20], particularly the lowest 80 m. The Purapura section is ~2 km up-valley of the approximate location Thouveny and Servant [8] give for their Purapura magnetostratigraphic section.

##### 1.1.5.2 Stratigraphy

**Table:** 

**Unit**	**Description**^**a**^	**Thickness (m)**
15	Weakly stratified, poorly sorted, clast-supported, pebble-cobble gravel (<10% granitic clasts)	2
14	Weakly stratified, poorly sorted, matrix-supported pebble-cobble diamicton (<10% granitic clasts)	6
	*Strong paleosol*^b^	
13	Massive, poorly sorted, matrix-supported pebble-cobble diamicton (<10% granitic clasts)	>2
–	Covered	12
12	Weakly stratified, matrix-supported pebble-cobble-boulder diamicton	>12
–	Covered	22
11	Stratified, poorly sorted, clast-supported, pebble-cobble gravel (<20% granitic clasts)	>6
10	Massive, matrix-supported, pebble-cobble-boulder diamicton with silt lenses	>5
–	Not sampled or systematically described	125
9	Weakly horizontally stratified, clast-supported, pebble-cobble-boulder gravel (90% granitic clasts)	>7
8	Massive, matrix-supported pebble-cobble diamicton (50% granitic clasts)	0.5
	*Strong paleosol*^b^	
7	Massive, matrix-supported pebble-cobble diamicton (50% granitic clasts)	3.5
	*Strong paleosol*^b^	
6	Massive, matrix-supported, pebble-cobble-boulder diamicton (<50% granitic clasts)	30.5
5	Weakly stratified, matrix-supported, pebble- cobble diamicton (50% granitic clasts)	11
4	Weakly laminated silt and sand	4
3	Rhyolitic tuff	5
2	Tuffaceous silt and sand with current structures; sharp lower contact	1.5
1	Weakly stratified, matrix-supported, pebble-cobble gravel (90% granitic clasts)	1.5

^a^All diamicton units contain striated and faceted clasts; most clasts are subrounded to subangular. Most clasts in gravel units are rounded to subrounded.

^b^The paleosols are classified as either strong or weak, depending on the degree of development of soil horizons and pedogenic structure. Strong paleosols are those with B horizons thicker than 50 cm (e.g. Fig. 5C of Ref. [14]) and pedons coated with clay (e.g. Fig. 5I of Ref. [14]). In contrast, weak paleosols are those with B horizons thinner than 30 cm, and little or no clay translocation and pedon formation.

#### 1.1.6 Jacha Kkota

##### 1.1.6.1 Location

The Jacha Kkota section (16° 34.67′ S, 68° 10.25′ W, 175 m in height; Table 7; Fig. 6F of Ref. [14]) is exposed in the gullies of a ridge west of Laguna Jacha Kkota, rising from the floor of the Achocalla basin to the Altiplano surface (Fig. 1C). The ridge is an intact remnant of the fill sequence below the Altiplano, which was left behind when a gigantic, early Holocene earthflow created the Achocalla basin [23] shortly before 11,485–10,965 cal yr BP [24]. The section includes the Chijini Tuff and 42 m of fine-grained sediments directly below it. The Achocalla magnetostratigraphic section of Thouveny and Servant [8] extends from the base of the tuff 80 m upslope along the same ridge, but does not reach the Altiplano surface.

We included the magnetostratigraphy of the uppermost 10 m of the sedimentary sequence at a nearby section (~3.2 km to the southeast); Fig. 1 to extend the Jacha Kkota section to the Altiplano surface. The exact stratigraphic alignment of the two sections is uncertain, but in view of the similar elevations of the Altiplano surface and the Chijini Tuff at both sites, the upper part of the sequence probably starts 40–45 m above the top of Thouveny and Servant's [8] Achocalla section, in agreement with the stratigraphy of the Achocalla basin margins reported by Bles et al. [19] and Ballivián et al. [20].

##### 1.1.6.2 Stratigraphy

**Table:** 

**Unit**	**Description**^**a**^	**Thickness (m)**
8	Poorly sorted, clast-supported, pebble-cobble gravel	3.5
	*Strong paleosol*^b^	
7	Weakly stratified, matrix-supported, pebble-cobble diamicton	7
	Not sampled or systematically described	42
6	Interbedded silt and sand (described by Thouveny and Servant [8] as clay and silt)	31
5	Rhyolitic tuff	5.5
4	Interbedded silt and silty sand	19
	*Strong paleosol*^b^	
3	Silt and underlying cross-bedded, medium to coarse sand; erosional basal contact	5
2	Interbedded silt, sand, and pebble gravel; erosional basal contact	30
1	Weakly laminated fine sandy silt	10

^a^All diamicton units contain striated and faceted clasts; most clasts are subrounded to subangular. Most clasts in gravel units are rounded to subrounded.

^b^The paleosols are classified as either strong or weak, depending on the degree of development of soil horizons and pedogenic structure. Strong paleosols are those with B horizons thicker than 50 cm (e.g. Fig. 5C of Ref. [14]) and pedons coated with clay (e.g. Fig. 5I of Ref. [14]). In contrast, weak paleosols are those with B horizons thinner than 30 cm, and little or no clay translocation and pedon formation.

### 1.2 Paleomagnetic data

 See Table 2, Table 3, Table 4, Table 5, Table 6, Table 7.

**Table 2:** Means of paleomagnetic directional data for the Patapatani West section.

**Unit**	**Lithology**		**χ**	**n**			***D***	***I***	***k***	***α***_***95***_^a^	**p**^a^
**Position (m)**	**Material**		**(mean)**	**Collected**	**Useful**	**Used**					
**PTW-20**	**Altiplano gravel**			Not sampled							
**PTW-19**	**Sorata Drift**										
230.5	Paleosol		779*	12	12	12	1.0	−27.0	106.03	4.2	N
220.5			189	6	6	5	3.2	−33.1	65.26	9.5	N
220.0			213	6	6	6	5.2	−36.3	71.78	8.0	N
214.5			294	6	6	5	333.6	−35.5	79.45	8.6	N
				**30**	**30**	**28**	**357.7**	**−32.1**	**37.53**	**4.5**	**N**
**PTW-18**	**Kaluyo Gravels**										
213.5			431	6	6	6	17	−32.1	49.49	9.6	N
											**N**
**PTW-17**	**Kaluyo Gravels**										
187.5			299	12	12	10	173.6	25.8	14.70	13.0	R
											**R**
**PTW-16**	**Purapurani Gravels**										
183.5	Sand lens		114	6	6	6	174.0	20.1	75.79	7.7	R
											**R**
**PTW-15**	**Calvario Drift**										
176.0	Diamict		73	6	0	0	Indeterminate polarity				−
175.5	Silt bed		104	6	2	2	222.5	45.3	--	--	R
				**12**	**2**	**2**					**R**
**PTW-14**	**Calvario Drift**										
165.5	Paleosol		1054*	6	6	6	152.3	55	18.53	16	R
162.0	Silt bed		700	3	3	3	161.4	37.0	319.48	6.9	R
				**9**	**9**	**9**	**156.1**	**48.9**	**20.81**	**11.6**	**R**
**PTW-13**	**Calvario Drift**										
161.0	Paleosol		2455*	3	3	3	348.6	−35.7	105.54	12.1	N
											**N**
**PTW-12**	**Calvario Drift**										
153.0	Silt bed (likely ash)		2532	3	3	3	357.7	−22.3	35.92	20.9	N
											**N**
**PTW-11**	**Calvario Drift**										
152.5	Paleosol		1633*	3	3	3	353.7	−42.9	228.28	8.2	N
143.0	Sand lens		386	6	6	6	3.7	−35.2	289.36	3.9	N
139.5	Silt/sand lense		168	6	6	6	350.5	−3.5	10.59	21.6	N
134.0	Diamict & silt lens		164	12	11	8	43.3	−22.2	21.73	12.2	N
114.5	Silt lens		154	6	6	5	346.4	−47.2	30.27	14.1	N
109.5	Silt lens		181	6	6	6	12.6	−38.1	34.75	11.5	N
109.0	Diamict		138	6	6	5	331.0	−62.7	124.93	6.9	N
108.0	Sand lens		180	6	6	6	354.9	−28.8	45.05	10.1	N
107.5	Sand lens		151	6	1	1	356.5	−46.8	−	−	N
				**57**	**51**	**46**	**4.8**	**−35.4**	**8.82**	**7.6**	**N**
**PTW-10**	**Chijini Tuff**										
103.5	Cliff-forming ash		1509^b^	6	6	6	18.3	−38.6	120.90	6.1	N
101.0	Cliff-forming ash		573	6	6	6	10.8	−37.2	90.25	7.1	N
100.0	Cliff-forming ash		686	6	6	3	0.9	−27.1	364.37	6.5	N
98.0	Cliff-forming ash		846	6	6	5	10.3	−29.8	82.66	8.5	N
97.0	Cliff-forming ash		729	6	6	6	15.6	−28.7	69.20	8.1	N
95.5	Silt-sized ash		407	6	6	5	6.7	−44.1	70.09	9.1	N
				**36**	**36**	**31**	**11.5**	**−34.9**	**56.79**	**3.5**	**N**
**PTW-09**	**Patapatani Drift**										
94.5	Diamict		119	6	6	6	0.5	−13.2	37.02	11.2	N
92.0	Diamict		74	6	6	6	8.3	−29.6	28.04	12.9	N
91.0	Diamict		284	6	6	6	355.9	−36.9	65.83	8.3	N
				**18**	**18**	**18**	**1.7**	**−26.7**	**24.15**	**7.2**	**N**
**PTW-08**	**Patapatani Drift**										
89.5	Paleosol formed in diamict		2368*	6	6	6	6.0	−43.6	250.61	4.2	N
88.0	Diamict		85	6	6	5	28.4	−27.4	20.66	17.2	N
				**12**	**12**	**11**	**17.1**	**−36.9**	**22.21**	**9.9**	**N**
**PTW-07**	**Patapatani Drift**										
85.5	Paleosol formed in diamict		5556*	6	6	6	19.8	−28.0	343.83	3.6	N
81.5	Diamict		155	6	4	3	347.7	−23.2	66.73	15.2	N
				**12**	**10**	**9**	**9.0**	**−27.2**	**26.55**	**10.2**	**N**
**PTW-06**	**Patapatani Drift**										
77.5	Paleosol formed in diamict		2641*	6	6	4	182.4	42.5	82.43	10.2	R
74.5	Diamict		125	6	6	5	166.2	14.6	143.25	6.4	R
70.0	Sand lens		168	6	4	3	217.0	55.6	41.51	19.4	R
62.0	Silt lens		162	6	6	4	182.7	1.6	31.32	16.7	R
				**24**	**22**	**16**	**180.7**	**26.8**	**9.17**	**12.9**	**R**
**PTW-05**	**Patapatani Drift**										
57.5	Paleosol (weakly developed)		154	6	4	4	213.3	70.0	7.93	34.8	R
52.5	Silt lens		100	14	9	6	187.6	28.8	19.82	15.4	R
				**20**	**13**	**10**	**192.6**	**44.9**	**7.09**	**19.5**	**R**
**PTW-04**	**Patapatani Drift**										
38.0	Silt lens		149	8	8	6	356.3	−28.4	55.67	9.1	N
											**N**
**PTW-03**	**Patapatani Drift**										
19.0	Silt lens		127	12	9	9	191.1	26.0	7.80	19.7	R
9.5	Sand lens & diamict		146	16	12	10	164.9	67.5	30.58	8.9	R
				**28**	**21**	**19**	**182.2**	**49.6**	**6.88**	**13.8**	**R**
**PTW-02**	**Pre-Patapatani stratified diamict**										
7.0	Sand lens		293	6	6	6	358.2	−32.4	173.29	5.1	N
4.5	Silt lens & diamict		158	12	11	8	5.4	−15.7	30.89	10.1	N
				**18**	**17**	**14**	**2.5**	**−23.0**	**30.88**	**7.3**	**N**
**PTW-01**	**Pre-Patapatani gravels**										
1.5	Gravel matrix		113	6	5	4	359.1	−15.2	14.93	24.6	N
											**N**

See Fig. 6A of Ref. [14] for stratigraphy and stratigraphic positions of sample groups. Position, sampling height in metres above base of section; *χ*, mean magnetic susceptibility of collected samples (×10^−6^ SI units); *n*, number of samples; *D* and *I*, mean declination and inclination, respectively; *k*, precision parameter; *α*_95_, circle of confidence (*P*=0.05); p, polarity.

* Magnetic enhancement of paleosol compared to the parent material in which it formed.

a Error between 10° and 20° underlined; error greater than 20° double underlined.

b Apparent magnetic enhancement at top of tuff unit.

**Table 3:** Means of paleomagnetic directional data for the Patapatani East section.

**Unit**	**Lithology**	***χ***	***n***			***D***	***I***	***k***	***α***_***95***_^a^	**p**^a^
**Position (m)**	**Material**	**(mean)**	**Collected**	**Useful**	**Used**					
**PTE-08**	**Calvario Drift**									
39.0	Fine sand lens	344	6	6	6	349.8	−24.1	75.99	7.7	N
										**N**
**PTE-07**	**Calvario Drift**									
37.0	Silt lens	206	6	6	4	339.2	−32.3	56.31	12.4	N
										**N**
**PTE-06**	**Chijini Tuff**									
31.5	Pumacious ash	636	6	6	6	359.7	−38.2	159.30	5.3	N
										N
**PTE-05**	**Patapatani Drift**		Not sampled							
**PTE-04**	**Patapatani Drift**									
28.0	Silt lens	130	12	12	10	10.9	−33.2	26.38	9.6	N
			**12**	**12**	**10**					**N**
**PTE-03**	**Patapatani Drift**									
	Paleosol		Not sampled							
**PTE-02**	**Patapatani Drift**									
6.5	Silt lens	152	6	6	5	146.9	19.4	45.93	11.4	R
										**R**
**PTE-01**	**Patapatani Drift**									
2.5	Fine sand lens	131	6	6	6	185.0	19.7	24.84	13.7	R
2.5	Fine sand lens	131	6	6	4	211.2	32.3	162.56	7.2	R
			**12**	**12**	**10**	**194.6**	**25.4**	**19.03**	**11.4**	**R**

See Fig. 6B of Ref. [14] for stratigraphy and stratigraphic positions of sample groups. Position, sampling height in metres above base of section; *χ*, mean magnetic susceptibility of collected samples (×10^−6^ SI units); *n*, number of samples; *D* and *I*, mean declination and inclination, respectively; *k*, precision parameter; *α*_95_, circle of confidence (*P*=0.05); p, polarity.

a Error between 10° and 20° underlined; error greater than 20° double underlined.

**Table 4:** Means of paleomagnetic directional data for the Tangani section.

**Unit**	**Lithology**	***χ***	***n***			***D***	***I***	***k***	***α***_***95***_^a^	**p**^a^
**Position (m)**	**Material**	**(mean)**	**Collected**	**Useful**	**Used**					
**TNG-13**	**Valley-slope cover**^b^									
Variable	Gravel matrix	76	6	6	3	328.0	−28.9	18.01	29.9	N
										**N**
**TNG-12**	**Purapurani Gravel**									
193.0	Silt bed	112	6	6	4	175.2	30.7	112.72	8.7	R
186.0	Silt bed	128	6	6	5	180.7	28.8	49.9	10.9	R
183.5	Silt lens	271	6	6	6	180.1	32.5	52.8	9.3	R
			**18**	**18**	**15**	**179.0**	**30.8**	**64.70**	**4.8**	**R**
**TNG-11**	**Purapurani Gravel**									
179.0	Gravel matrix	286	6	6	6	349.0	−18.6	60.42	8.7	N
										**N**
**TNG-10**	**Purapurani Gravel**									
158.0	Sand lens	439	6	6	6	165.8	25.6	89.57	7.1	R
142.0	Sand lens	76	6	6	5	175.8	39.8	92.62	8.0	R
			**12**	**12**	**11**	**169.9**	**32.1**	**47.42**	**6.7**	**R**
**TNG-9**	**Purapurani Gravel**									
141.0	Sand lens	1248*	6	6	6	138.1	43.0	72.84	7.9	R
129.0	Sand lens	23	6	6	4	167.2	21.3	95.66	9.4	R
			**12**	**12**	**10**	**151.5**	**35.2**	**18.69**	**11.5**	**R**
**TNG-08**	**Calvario Drift, diamict**									
127.5	Clay lens & diamict matrix	403*	12	12	11	187.5	26.3	22.68	9.8	R
125.0	Silt lens	93	6	0	0	Indeterminate polarity	–			
			**18**	**12**	**11**	**187.5**	**26.3**	**22.68**	**9.8**	**R**
**TNG-07**	**Calvario Drift, diamict**									
121.0	Diamict matrix	97	6	0	0	Indeterminate polarity				–
										–
**TNG-06**	**Calvario Drift, gravel**									
101.0	Silt lens	42	6	6	6	181.9	42.3	47.42	9.8	R
108.0	Fine-sandy silt bed	78	12	12	10	187.2	16.1	35.38	8.2	R
105.0	Silt & sand lens	46	6	6	6	177.8	24.6	46.60	9.9	R
102.5	Sand lens	85	9	9	8	173.9	19.4	42.18	8.6	R
			**33**	**33**	**30**	**180.7**	**23.9**	**25.01**	**5.4**	**R**
**TNG-05**	**Calvario Drift, diamict**									
101.0	Fine sand lens	173	6	6	6	177.7	24.9	245.95	4.3	R
105.0	Silt & sand lens	153	6	6	2	181.4	27.9	–	–	R
			**12**	**12**	**8**	**178.6**	**25.7**	**254.77**	**3.5**	**R**
**TNG-04**	**Calvario Drift, diamict**									
104.5	Sand bed	804*	6	6	5	342.9	−41.6	21.19	17.0	N
86.0	Silt lens	218	6	6	6	354.7	−11.4	76.11	7.7	N
74.0	Silt lens	218	6	6	5	336.3	−20.5	23.98	15.9	N
70.0	Silt lens	184	6	6	6	339.6	−18.6	62.97	8.5	N
66.0	Sand lens	113	5	5	5	4.5	−23.9	72.33	9.1	N
52.5	Silt lens	133	5	5	4	9.1	−37.4	43.95	14.0	N
49.0	Silt lens	169	6	6	6	0.3	−13.6	36.50	11.2	N
44.0	Silt lens	172	6	6	5	2.6	0.1	36.20	12.9	N
20.0	Silt lens	178	6	6	4	0.6	−2.8	37.83	15.1	N
			**52**	**52**	**46**	**354.1**	**−18.8**	**16.29**	**5.4**	**N**
**TNG-03**	**Calvario Drift, gravel**									
13.0	Silt lens	686	6	6	6	352.3	−29.7	85.02	7.3	N
										**N**
**TNG-02**	**Calvario Drift, diamict**									
8.0	Silt bed	165	6	6	6	346.5	−33.8	425.25	3.3	N
										**N**
**TNG-01**	**Calvario Drift, gravel**									
4.5	Silt lens	188	6	6	6	356.3	−19.0	46.47	9.9	N
2.5	Fine sand lens	284	6	6	5	3.8	−35.7	62.39	9.8	N
			**12**	**12**	**11**	**359.5**	**−26.2**	**32.95**	**8.1**	**N**

See Fig. 6C of Ref. [14] for stratigraphy and stratigraphic positions of sample groups. Position, sampling height in metres above base of section; *χ*, mean magnetic susceptibility of collected samples (×10^−6^ SI units); n, number of samples; *D* and *I*, mean declination and inclination, respectively; *k*, precision parameter; *α*_95_, circle of confidence (*P*=0.05); p, polarity.

* Magnetic enhancement of paleosol compared to the parent material in which it formed.

a Error between 10° and 20° underlined; error greater than 20° double underlined.

b Colluvium draping incised valley slope (possible mass flow deposit).

**Table 5:** Means of paleomagnetic directional data for the Minasa section.

**Unit**	**Lithology**	***χ***	***n***			***D***	***I***	***k***	***α***_***95***_^a^	**p**^a^
**Position (m)**	**Material**	**(mean)**	**Collected**	**Useful**	**Used**					
**MIN-15**	**Gravel grading to diamict**									
410.0	Modern soil (in diamict)	1354*	6	6	5	1.5	−33.7	183.39	5.7	N
										**N**
**MIN-14**	**Gravel grading to diamict**									
406.0	Paleosol (in sand lens)	1309*	6	6	6	354.9	−24.8	118.90	6.2	N
										**N**
**MIN-13**	**Diamict**									
402.5	Sand lens	323	6	6	6	6.1	−21.5	57.25	8.9	N
										**N**
**MIN-12**	**Gravel grading to diamict**									
378.0	Sand lens	111	6	6	6	350.7	−22.6	26.17	13.3	N
										**N**
**MIN-11**	**Gravel**					Not sampled				
**MIN-010**	**Gravel grading to diamict**					Not sampled				
**MIN-09**	**Gravel grading to diamict**					Not sampled				
**MIN-08**	**Diamict**									
324.0	Sand lens	125	6	6	5	356.1	−24.4	76.90	8.8	N
										**N**
**MIN-07**	**Gravel**					Not sampled				
**MIN-06**	**Diamict**					Not sampled				
**MIN-05**	**Kaluyo gravel & Sorata Drift**									
324.0	Silt lens	91	6	5	4	350.3	−24.8	52.15	12.8	N
										**N**
**MIN-04**	**Kaluyo gravel & Sorata Drift**					Not sampled				
**MIN-03**	**Purapurani Gravel**									
278.0	Silt bed	337	6	0	0	Indeterminate polarity				–
275.0	Silt bed	71	6	6	4	187.3	31.2	50.14	13.1	R
234.0	Silty sand lens	71	6	6	6	176.6	38.9	65.88	8.3	R
214.0	Sand bed	82	6	6	4	176.8	40.0	73.25	10.8	R
			**24**	**18**	**14**	**179.9**	**37.1**	**54.77**	**5.4**	**R**
**MIN-02**	**Multi-lithic gravel**									
144.0	Paleosol (in gravel)	1926*	6	6	5	143.7	38.2	92.85	8.0	R
112.0	Silt lens	102	6	6	4	175.8	24.7	211.71	6.3	R
10.5	Medium sand lens	107	6	0	0	Indeterminate polarity				–
			**18**	**12**	**9**	**159.1**	**33.2**	**21.91**	**11.2**	**R**
										
0.0	Silt-sized ash	85	6	6	6	259.6	−35.8	28.91	12.7	N
										**N**

See Fig. 6D of Ref. [14] for stratigraphy and stratigraphic positions of sample groups. Position, sampling height in metres above base of section; *χ*, mean magnetic susceptibility of collected samples (×10^−6^ SI units); n, number of samples; *D* and *I*, mean declination and inclination, respectively; *k*, precision parameter; α_95_, circle of confidence (*P*=0.05); p, polarity.

* Magnetic enhancement of paleosol compared to the parent material in which it formed.

a Error between 10° and 20° underlined; error greater than 20° double underlined.

**Table 6:** Means of paleomagnetic directional data for the Purapura section.

**Unit**	**Lithology**	***χ***	***n***			***D***	***I***	***k***	***α***_***95***_^a^	**p**^a^
**Position (m)**	**Material**	**(mean)**	**Collected**	**Useful**	**Used**					
**PUR-15**	**Altiplano surface gravels**					Not sampled				
**PUR-14**	**Sorata Drift, diamicton**									
246	Silt lens	210	8	8	8	345.8	−25.0	112.37	5.2	N
244	Silt lens	525	6	6	6	1.0	−29.0	317.46	3.8	N
			14	14	14	**352.2**	**−26.9**	**71.56**	**4.7**	**N**
**PUR-13**	**Sorata Drift, diamicton**									
240	Palsosol	1461*	8	8	8	358.0	−30.2	230.97	3.7	N
										**N**
**PUR-12**	**Sorata Drift, diamicton**					Not sampled				
**PUR-11**	**Sorata Drift, gravel**					Not sampled				
**PUR-10**	**Sorata Drift, diamicton**									
192		121	6	6	6	351.6	−21.6	48.68	9.7	N
**PUR-09**	**Purapurani Gravel**									
58.5		56	6	**0**	**0**	Indeterminate polarity				–
**PUR-08**	**Calvario Drift**									
58		115	6	5	4	30.1	−39.5	39.84	14.7	N
**PUR-07**	**Calvario Drift**									
57.5	Palsosol	342	6	6	4	3.2	−38.8	60.38	11.9	N
57.5	Palsosol	417	6	6	6	343.5	−53.3	24.14	13.9	N
			**12**	**12**	**10**	**352.8**	**−47.9**	**22.66**	**10.4**	**N**
**PUR-06**	**Calvario Drift**									
54	Palsosol	1631*	3	3	3	351.2	−28.9	401.54	6.2	N
23.5	Possible ash	1360*	7	7	6	337.5	−16.4		1.5	N^b^
			10	10	9		Mix of PCA and GC			**N**
**PUR-05**	**Calvario Drift**									
13.5		84	6	0	0		Indeterminate polarity			–
										**–**
**PUR-04**	**Calvario Drift, fines**					Not sampled				
**PUR-03**	**Chijini Tuff**									
3.5	Tuff	1727	6	6	6	357.6	−54.9	72.68	7.9	N
										**N**
**PUR-02**	**La Paz Formation, possible pyroclastic flow**									
2.5	Silt bed	102	3	3	3	16.1	−29.5	234.82	8.1	N
2	Silt bed	91	6	6	6	355.4	−30.2	281.72	4.0	N
			**9**	**9**	**9**	**2.3**	**−30.3**	**65.00**	**6.4**	**N**
**PUR-01**	**La Paz Formation, gravel**									

See Fig. 6E of Ref. [14] for stratigraphy and stratigraphic positions of sample groups. Position, sampling height in metres above base of section; *χ*, mean magnetic susceptibility of collected samples (×10^−6^ SI units); *n*, number of samples; *D* and *I*, mean declination and inclination, respectively; *k*, precision parameter; *α*_95_, circle of confidence (*P*=0.05); p, polarity.

* Magnetic enhancement of paleosol compared to the parent material in which it formed.

a Error between 10° and 20° underlined; error greater than 20° double underlined.

b Remanence directions obtained by the intersection of great circles.

**Table 7:** Means of paleomagnetic directional data for the Jacha Kkota section.

**Unit**	**Lithology**	***χ***	***n***			***D***	***I***	***k***	***α***_***95***_^a^	**p**^a^
**Position (m)**	**Material**	**(mean)**	**Collected**	**Useful**	**Used**					
**JKT-99**	**Altiplano surface gravel**									
178.0	Silt lens	194	6	6	5	355.4	−26.4	129.44	6.8	N
										**N**
**JKT-98**	**Diamicton**									
175.0	Silt lens	133	6	6	4	352.4	−7.4		7.9	N^b^
										**N**
**JKT-06**	**upper La Paz Formation**					Not sampled				
**JKT-05**	**Chijini Tuff**									
43.5	Cemented tuff	2122	6	6	6	357.0	−37.3	108.48	6.5	N
42.5	Loose ash	197	6	6	6	2.3	−28.9	258.18	4.2	N
			**12**	**12**	**12**	**359.8**	**−33.1**	**103.10**	**4.3**	**N**
**JKT-04**										
39.5	Silt bed	108	6	6	4	349.1	−36.8	1239.45	2.6	N
32.5	Fine sand bed	280	6	6	6	344.6	−26.1	53.91	9.2	N
			**12**	**12**	**10**	**348.3**	**−30.4**	**63.27**	**6.1**	**N**
**JKT-03**	**upper La Paz Formation**									
22.0	Paleosol	565*	6	6	5	354.7	−34.5	49.29	9.6	N
										**N**
**JKT-02**	**upper La Paz Formation**									
13.0	Silt lens	262	6	6	5	353.7	−23.2	98.05	8.2	N
										**N**
**JKT-01**	**upper La Paz Formation**									
5.5	Silt bed	157	6	5	4	187.9	38.2	95.20	9.5	R
										**R**

See Fig. 6F of Ref. [14] for stratigraphy and stratigraphic positions of sample groups. Position, sampling height in metres above base of section; *χ*, mean magnetic susceptibility of collected samples (×10^−6^ SI units); n, number of samples; *D* and *I*, mean declination and inclination, respectively; *k*, precision parameter; *α*_95_, circle of confidence (*P*=0.05); p, polarity.

* Magnetic enhancement of paleosol compared to the parent material in which it formed.

a Error between 10° and 20° underlined; error greater than 20° double underlined.

b Remanence directions obtained by the intersection of great circles.

## 2 Experimental design, materials and methods

### 2.1 Lithostratigraphic characterization

**Fig. 2:**
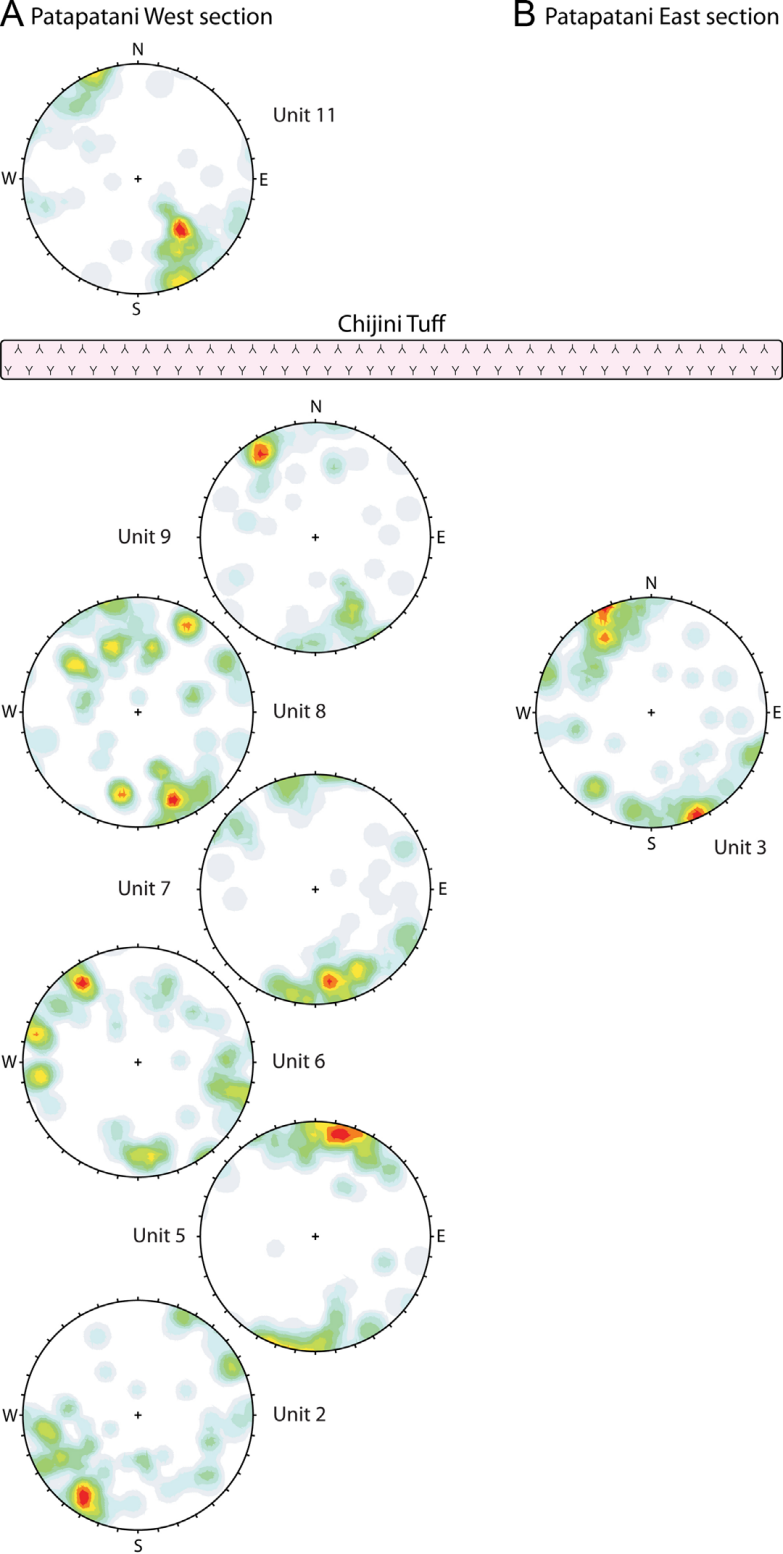
Diamicton clast fabrics from the (A) Patapatani West and (B) Patapatani East sections. The fabrics are each based on trends and plunges of 50 elongate pebbles and cobbles (long:short axis ratio of ≥2:1) and are represented by Fisher distributions on equal-angle stereonets. Orientation densities range from 0% (white) to 10% (red).

We measured and described six sections along the western margin of the La Paz and Achocalla basins, totaling 1100 vertical metres of exposure of the sediment sequence underlying the Altiplano plateau (Table 1). The sections are exposed in steep valley slopes, gullies, and road cuts and form a ~20-km-long transect through the eastern Altiplano margin, oblique to the trend of the Central Andes (Fig. 1). Sedimentologic and stratigraphic characterization include texture, structure, lithology, colour, clast size and shape, sorting, weathering features, and the nature of contacts. We divided units on the basis of major changes in material properties and on the occurrence of major hiatuses indicated by paleosols or erosional contacts. We measured unit thicknesses using a TruPulse 200 Laser Range Finder, and stratal thicknesses and sizes of clasts using a graduated metric scale. We measured the long-axes orientations (trend and plunge) of 50 elongate clasts from each of eight units at the two sections closest to the Cordillera Real (seven units at the Patapatani West section and one unit at the Patapatani East section: Fig. 2).

### 2.2 Materials

The sample collection comprises 808 oriented cylindrical samples (2.1 cm diameter, 1.8 cm length) collected typically in groups of six (ranging from three to 16) from 124 stratigraphic levels at the sections (Table 1). Sampling gaps due to limited exposure, inaccessibility, or unsuitably coarse sediments were filled where possible by sampling closely aligned units at nearby exposures. We collected larger numbers of samples in gravel and diamicton units to provide a more complete magnetostratigraphic record; coarse units are more likely to yield problematic paleomagnetic results [25] and are thus less commonly sampled in magnetostratigraphic studies, including the only previous paleomagnetic study in the La Paz area [8]. During subsequent field visits, we re-sampled sites that produced indeterminate polarity or incoherent magnetization characteristics.

Samples were typically taken in horizontally bedded zones of predominantly silt and fine to medium sand. Where these were not available, we collected samples from the matrices of gravel and diamicton units, avoiding granules and pebbles. Where possible, sampling included material both above and below unit boundaries. Samples were stored in magnetic shields at the University of Lethbridge following transport from the field and between measurements.

### 2.3 Magnetic susceptibility

Prior to demagnetization, we measured bulk magnetic susceptibility of each sample with a Sapphire Instruments SI-2B magnetic susceptibility meter.

### 2.4 Magnetic remanence

We measured natural remanent magnetization of each sample with an AGICO JR-6A spinner magnetometer. We re-measured remanence after stepwise alternating field (AF) demagnetization with an ASC Scientific D-2000 alternating-field demagnetizer in fields up to 200 mT. One or two pilot samples, having either representative or relatively high magnetic susceptibility, were selected from each group. These pilots were demagnetized at 10 to 16 closely spaced steps (intervals of 2.5–10 mT up to 80 mT, and 10–30 mT above 80 mT). The remaining samples from each group were then demagnetized at 4 to 10 steps (5–30 mT spacing) guided by characteristic magnetizations of pilot samples. Each sample was demagnetized to 20% or less of the natural remanent magnetization. Median destructive fields for most samples range from 10 to 80 mT, although a small number of samples included hard components of magnetization that remained following demagnetization at 200 mT AF (the limit of the equipment used).

We determined remanence directions for most samples by principal component analysis [26] and for a small number of samples (<2%) by the intersection of great circles [27] (Table 1). We calculated mean remanence directions by group (Table 2, Table 3, Table 4, Table 5, Table 6, Table 7), stratigraphic unit (Table 2, Table 3, Table 4, Table 5, Table 6, Table 7), and polarity (Table 1 and Fig. 3 of Ref. [14]). Sample-specific and mean remanence directions were calculated using AGICO's Remasoft v. 3.0.
